# Effects of an Aesthetics‐Based Educational Intervention on Compassion Competence and Aesthetic Care Quality Among Psychiatric Nurses: A Quasi‐Experimental Study

**DOI:** 10.1002/nop2.70854

**Published:** 2026-09-18

**Authors:** Donya Balideh, Jamileh Farokhzadian, Sakineh Miri, Sedigheh Khodabandeh Shahraki, Mohadeseh Motamed‐Jahromi

**Affiliations:** ^1^ Nursing Research Center Kerman University of Medical Sciences Kerman Iran; ^2^ Physiology Research Center Institute of Neuropharmacology, Kerman University of Medical Sciences Kerman Iran; ^3^ Student Research Committee Kerman University of Medical Sciences Kerman Iran; ^4^ Reproductive and Family Health Research Center Kerman University of Medical Sciences Kerman Iran; ^5^ Department of Public Health, School of Health Fasa University of Medical Sciences Fasa Iran

**Keywords:** compassion, educational programs, nursing care, nursing education, psychiatric nursing

## Abstract

**Aim:**

This study aimed to evaluate the effects of an aesthetics‐based educational intervention on compassion competence and aesthetic nursing care among psychiatric nurses.

**Design:**

A quasi‐experimental pretest–posttest study with a control group.

**Methods:**

The study was conducted at a psychiatric hospital in Kerman, Iran. A total of 90 eligible psychiatric nurses were randomly assigned to either an intervention group (*n* = 45) or a control group (*n* = 45). The intervention group participated in four weekly 90‐min aesthetics‐based nursing care educational sessions over four consecutive weeks, whereas the control group continued routine practice without receiving the educational intervention. Data were collected at baseline and 1 month after completion of the educational program using the Aesthetics of Nursing Care Scale (ENCS) and the Compassion Competence Scale (CCS). Data were analysed using descriptive and inferential statistics.

**Results:**

Following the intervention, the mean total aesthetic nursing care score increased significantly in the intervention group compared with baseline and the control group (81.02 ± 6.26 vs. 43.26 ± 2.18, *p* < 0.001). In contrast, no significant change was observed in the control group (*p* = 0.11). Similarly, the mean total compassion competence score increased significantly in the intervention group after the intervention (60.73 ± 16.17) compared with baseline and the control group (*p* < 0.001), while no significant change was found in the control group (*p* = 0.12).

**No Patient or Public Contribution:**

No patient or public contribution was involved in this study.

## 1 Introduction

Nurses are primary healthcare providers whose role is crucial in psychiatric wards, where they face unique challenges such as managing mental disorders (Navvab et al. 2022). Recently, physical stressors and demanding environments have increasingly compromised the quality of care and the efficacy of therapeutic relationships in these settings (Sargazi et al. 2018b; Sarvarizadeh et al. 2024). To address these challenges, the concepts of ‘compassion’ and ‘aesthetics’ have been proposed for enhancing nursing practice (Fesharaki and Radmehr 2020). The aesthetic model in nursing, often referred to as the ‘art of nursing’, emphasizes empathy and compassion as fundamental clinical skills (Allande‐Cussó et al. 2022; Farokhzadian et al. 2025; Mafakheri et al. 2024).

Beyond routine care, aesthetic philosophy utilizes artistic expression and sensory experiences to illuminate both concrete and abstract aspects of patient care, fostering meaningful interactions (Li et al. 2025; Vega and Hayes 2019). Researchers stress that it is now time to examine the aesthetics and art of nursing, which go hand in hand with knowledge, dedication, spirituality, and kindness (Li et al. 2025). This approach promotes creativity, flexibility, and a holistic perspective, preventing nursing from becoming a routine process. By acknowledging patients in their entirety and utilizing innovative resources, nurses can alleviate discomfort and foster a positive therapeutic atmosphere (Betriana et al. 2022; Shahmohammadi et al. 2024).

Complementing aesthetics, compassion is a cornerstone of patient‐centered care. It requires a profound understanding of the patient's needs and a commitment to alleviating their suffering (Mohammed et al. 2024). Compassion competence (CC) integrates this moral attribute with clinical excellence, involving the judicious application of theoretical knowledge, communication skills, and emotional intelligence in daily practice (Hong and Han 2021; Ilarde et al. 2021; Niroomandan and Ahi 2020). Furthermore, aesthetic‐based care education facilitates a profound understanding of aesthetics and its pivotal role in elevating nursing quality. By analysing the psychological and emotional dimensions of care through lived narratives and clinical experiences, this pedagogical approach fosters a sense of solidarity and oneness between the nurse and the patient (Khooshab et al. 2024; Shahmohammadi et al. 2024).

Several studies have underscored the necessity of aesthetic care training to foster a better understanding of nursing practice. This approach aims to improve nursing performance, reduce stress, boost motivation, and enhance patient cooperation during recovery. Furthermore, strengthening CC has been proposed as a strategy to improve overall caring behaviours (Kongsuwan 2020; Thompson 2022). Evidence suggests that implementing aesthetics‐based care positively influences the quality of clinical practice and contributes to the professional advancement of nursing (Li et al. 2025; Shahmohammadi et al. 2024). Specifically, aesthetic experiences serve as valuable tools for addressing existential distress and the sense of meaninglessness often associated with severe illness (Miller et al. 2023). Recent findings indicate a correlation between aesthetics‐based care and compassion competence, suggesting that nurses who engage in aesthetics‐based care exhibit higher levels of compassion than their peers. Consequently, incorporating aesthetics into nursing curricula has the potential to enhance the quality of compassionate nursing care (Betriana et al. 2022; Fesharaki and Radmehr 2020).

Despite the recognized importance of compassion, competence, and aesthetics‐based care, research within the specialized field of psychiatric nursing remains limited. This gap is particularly important because psychiatric patients frequently experience impaired communication, emotional dysregulation, social isolation, stigma, and difficulties establishing trusting therapeutic relationships (Prizeman et al. 2023). Consequently, psychiatric nursing relies heavily on empathy, therapeutic communication, emotional presence, and compassionate interpersonal interactions (Gee 2025). Nurses who provide compassionate and aesthetically grounded care may be better able to establish therapeutic relationships and engage patients in the recovery process (Ghafourifard et al. 2022). These approaches have also been associated with improvements in the quality of mental health care. Moreover, the complexity of mental disorders, the need for specialized interventions, and the demanding nature of psychiatric care make the implementation of these approaches particularly challenging in psychiatric settings (Hasan et al. 2018). Deficits in these competencies may contribute to nurse burnout, reduced job satisfaction, interpersonal isolation, and emotional exhaustion, thereby limiting the personal resources required to provide high‐quality psychiatric nursing care (Mahler 2019).

Despite these challenges, evidence regarding educational interventions aimed at improving aesthetic nursing care and compassion competence among psychiatric nurses remains scarce. Although previous educational interventions have demonstrated that structured training programs can improve the professional competencies of psychiatric nurses (Sargazi et al. 2018a), little is known about the effectiveness of aesthetics‐based educational approaches in enhancing compassion competence and aesthetic nursing care. Therefore, this study aimed to address this knowledge gap by investigating the effect of an aesthetics‐based educational program on compassion competence and aesthetic nursing care among psychiatric nurses in Kerman, Iran.

## 2 Methods

### 2.1 Study Type and Setting

This study employed a quasi‐experimental pretest–posttest design with a control group and was conducted at Shahid Beheshti Psychiatric Hospital, the largest educational and referral center for mental health disorders in southeastern Iran. Although participants were randomly allocated to the study groups, blinding was not feasible because of the educational nature of the intervention. Consequently, neither participants nor researchers were blinded to group allocation. Therefore, despite the use of random allocation, the study was designed as a single‐center quasi‐experimental study rather than a randomized controlled trial. This study is reported in accordance with the Transparent Reporting of Evaluations with Nonrandomized Designs (TREND) statement (Des Jarlais et al. 2004), and the completed TREND checklist is provided in the File S1.

At the time of the study, approximately 170 registered psychiatric nurses were employed at the hospital. Of these, 90 eligible nurses met the inclusion criteria and consented to participate in the study. Participants were randomly assigned to the intervention or control group (*n* = 45 per group) using a lottery‐based simple random allocation method. Simple random allocation was used to minimize selection bias and to improve baseline comparability between the two study groups. Each eligible participant drew a coded lot corresponding to either the intervention or control group, ensuring an equal probability of allocation. The individual nurse served as both the unit of assignment and the unit of analysis. The sample size was calculated as 45 participants per group based on a 95% confidence level and 80% statistical power, using the methodology described in a previous study (Shahmohammadi et al. 2024).

The inclusion criteria were: providing written informed consent, self‐reported absence of psychotic symptoms, no concurrent psychological treatment during the study period, holding a bachelor's degree or higher in nursing, and having at least 6 months of work experience in a psychiatric ward. The exclusion criteria were: absence from more than 10% of the educational sessions, leave of absence or transfer to another hospital during the study period, and submission of incomplete questionnaires (defined as more than 5% of questionnaire items left unanswered). Attendance was monitored throughout the intervention, and all participants attended the scheduled educational sessions in accordance with the study protocol.

### 2.2 Data Collection Tools

Data collection tools included a demographics form, the Aesthetics of Nursing Care Scale (ENCS), and the Compassion Competence Scale (CCS).

### 2.3 Demographic Questionnaire

The questionnaire comprised items on demographic and professional characteristics, including age, gender, marital status, educational background, employment status, position, overall work experience, experience in psychiatric wards, shift type, and participation in aesthetic care and compassion training courses.

### 2.4 Aesthetic of Nursing Care Scale (ENCS)

The initial ENCS in Iran was developed by Radmehr et al. 2025, The research team validated the ENCS for application within the nursing community. Exploratory and confirmatory factor analyses led to the removal of seven items, resulting in a 20‐item scale structured across five subscales: compassionate commitment and competence (4 items), stress‐free care (4 items), humanistic attention to patient (3 items), patient satisfaction and comfort (5 items), and admirable commitment and competence (4 items). The response range of the scale includes never = 0, rarely = 1, sometimes = 2, often = 3, most of the times = 4, and always = 5. There are no inverse items in the scale. The minimum score of the scale is zero and the maximum score is 100. I‐CVI of 0.8 and the S‐CVI of ≥ 0.9 were acceptable. EFA showed that the subscales explained 74.5% of the total variance, a structure further validated by CFA. Convergent validity was confirmed due to a strong correlation between the ENCS and the compassion competence scale (*r* = 0.537). Additionally, the scale had good reliability (Cronbach's alpha coefficient = 0.956 and Omega coefficient = 0.957) (Radmehr et al. 2025).

### 2.5 Compassion Competence Scale (CCS)

Lee and Seomun (2016) developed this scale as a self‐report instrument to assess nurses' CC. This instrument comprises 17 items organized into three subscales, including communication (eight items), sensitivity (five items), and insight (four items). Responses are measured on a scale from 1 (strongly disagree) to 5 (strongly agree). The minimum total score is 17, while the maximum total score is 85. The reliability of this tool, assessed using Cronbach's alpha coefficient, is estimated at 0.91 for the overall test, 0.88 for the communication, 0.77 for sensitivity, and 0.73 for insight subscales (Lee and Seomun 2016). In 2019, Niroomandan and Ahi in Iran used an exploratory factor analysis with 330 nurses to assess the validity of this scale, which explained 53.60% of the total variance of the test (Niroomandan and Ahi 2020).

The internal consistency was evaluated by correlating the overall CC score with its subscales, resulting in coefficients of 0.76, 0.77, and 0.93, respectively. The values indicate strong internal consistency for the scale. Exploratory factor analysis resulted in the elimination of one item (item 5), yielding a 16‐item questionnaire. The minimum and maximum achievable scores were thus 16 and 80. The reliability of the questionnaire was confirmed through test–retest (*r* = 0.78) and internal consistency (*α* = 0.85) methods, supporting its psychometric soundness (Niroomandan and Ahi 2020).

### 2.6 Intervention and Data Collection

Once the ethical approval and permission from the hospital authorities were obtained, the study was conducted at Shahid Beheshti Psychiatric Hospital. Eligible participants were informed about the study objectives, procedures, and ethical considerations, and written informed consent was obtained. Participants were randomly assigned to the intervention and control groups using a lottery‐based simple random allocation method. To minimize contamination between groups, the intervention and control groups were selected from separate wards, and participants in the intervention group were requested not to share the educational materials until the completion of the study. Data collection, including the demographic questionnaire, the ENCS, and the CCS, was conducted between June 2024 and March 2025.

Two nursing faculty members specializing in psychiatric nursing and aesthetics delivered the aesthetics‐based care training. The educational intervention consisted of four structured 90‐min face‐to‐face sessions delivered once a week over four consecutive weeks (see Table 1 for an overview of the session goals and content).

**TABLE 1:** Overview of the aesthetics‐based nursing care training program.

Sessions	Goals	Content of sessions
1	To introduce the concepts and foundations of aesthetics‐based nursing care	Introduction to the educational intervention, including its objectives, structure, and expected outcomes General and specific definitions of aesthetics, compassion, and the core competencies of compassion An overview of the historical development and theoretical foundations of aesthetics in nursing care, emphasizing its conceptualization as an art Discussion of the impact of aesthetics‐based nursing care on the quality of psychiatric nursing care Interactive group discussion and question‐and‐answer session
2	To educate nurses about the psychological dimensions of care and their role in promoting mental health through aesthetics‐based nursing care	Group discussion on the subjective nature of nursing aesthetics. Participants explored how touch, tone of voice, auditory cues, gestures, and deliberate word choice contribute to aesthetic nursing care. These concepts were illustrated through participants' lived experiences and reflective narratives (Allande‐Cussó et al. 2022; Shahmohammadi et al. 2024) Discussion of the spiritual dimension of aesthetics in nursing care and nurses' lived experiences Discussion of how aesthetics‐based nursing care can reduce patients' feelings of helplessness and anxiety while promoting comfort, security, and emotional well‐being through compassionate care and shared experiences Assignment: Participants were asked to reflect on and share their own aesthetic care experiences during the following session Educational videos and psychiatric clinical scenarios were presented
3	To strengthen nurses' sense of connectedness with patients and examine how professional skills and experiences contribute to aesthetics‐based nursing care	Discussion of the role of empathy and therapeutic companionship in improving communication, interpersonal relationships, and patient connectedness during aesthetics‐based nursing care, supported by participants' lived experiences Discussion of how nurses use creativity and professional commitment within an aesthetic framework to address clinical challenges and uncertainties Assignment: Participants were asked to discuss and share their own clinical experiences during the following session
4	To summarize the role of aesthetics in nursing care and reflect on nurses' learning experiences	Discussion of how aesthetics‐based nursing care enhances nurses' job satisfaction, collaboration, communication, and sense of inner peace, thereby strengthening professional motivation and retention Assignment: Participants were invited to share their reflections and relevant clinical experiences related to aesthetics‐based nursing care

Between sessions, participants completed reflective assignments and reflected on their aesthetic care experiences, which were discussed at the beginning of the subsequent session. The educational content was developed based on a comprehensive review of the literature on aesthetics‐based nursing care, compassion competence, and nursing education (Allande‐Cussó et al. 2022; Shahmohammadi et al. 2024) and was subsequently validated by a panel of seven experts (five faculty members and two senior psychiatric nurses). The sessions employed a variety of pedagogical methods, including lectures, question‐and‐answer sessions, group discussions, PowerPoint presentations, educational videos, and psychiatric‐specific scenario analyses. Nurses were divided into groups of five to complete homework assignments and share their lived experiences of aesthetic care during subsequent sessions. A comprehensive booklet summarizing the educational content was provided to participants in the intervention group.

The control group received routine practice without any educational intervention during the study period. 1 month after completion of the educational program, participants in both groups completed the post‐intervention questionnaires (ENCS and CCS). A 1‐month follow‐up was selected to assess the short‐term retention and application of the educational content in clinical practice rather than participants' immediate reactions after the final training session. This interval also reduced the likelihood that the observed effects merely reflected temporary post‐training enthusiasm. All completed questionnaires were reviewed immediately after collection by the principal investigator to ensure completeness and minimize missing data. After completion of data collection, an abbreviated version of the educational workshop was offered to the control group to ensure ethical fairness.

### 2.7 Data Analysis

Data were analysed using SPSS version 27. Descriptive statistics, including frequencies, percentages, means, and standard deviations, were used to summarize participants' characteristics and study variables. The normality of continuous variables was assessed using the Kolmogorov–Smirnov test. Baseline demographic characteristics were compared between the intervention and control groups using the Chi‐square test or Fisher's exact test for categorical variables, as appropriate, and the independent *t*‐test for continuous variables. Within‐group changes in aesthetic nursing care and compassion competence scores from pre‐test to post‐test were assessed using paired *t*‐tests, whereas between‐group differences at baseline and post‐intervention were evaluated using independent *t*‐tests. Effect sizes were calculated using Cohen's d and interpreted as small (0.20), medium (0.50), and large (0.80). A 2‐tailed *p*‐value < 0.05 was considered statistically significant. Because all participants completed both the baseline and post‐intervention assessments, no missing data imputation was required.

## 3 Results

### 3.1 Demographic Characteristics

**FIGURE 1:**
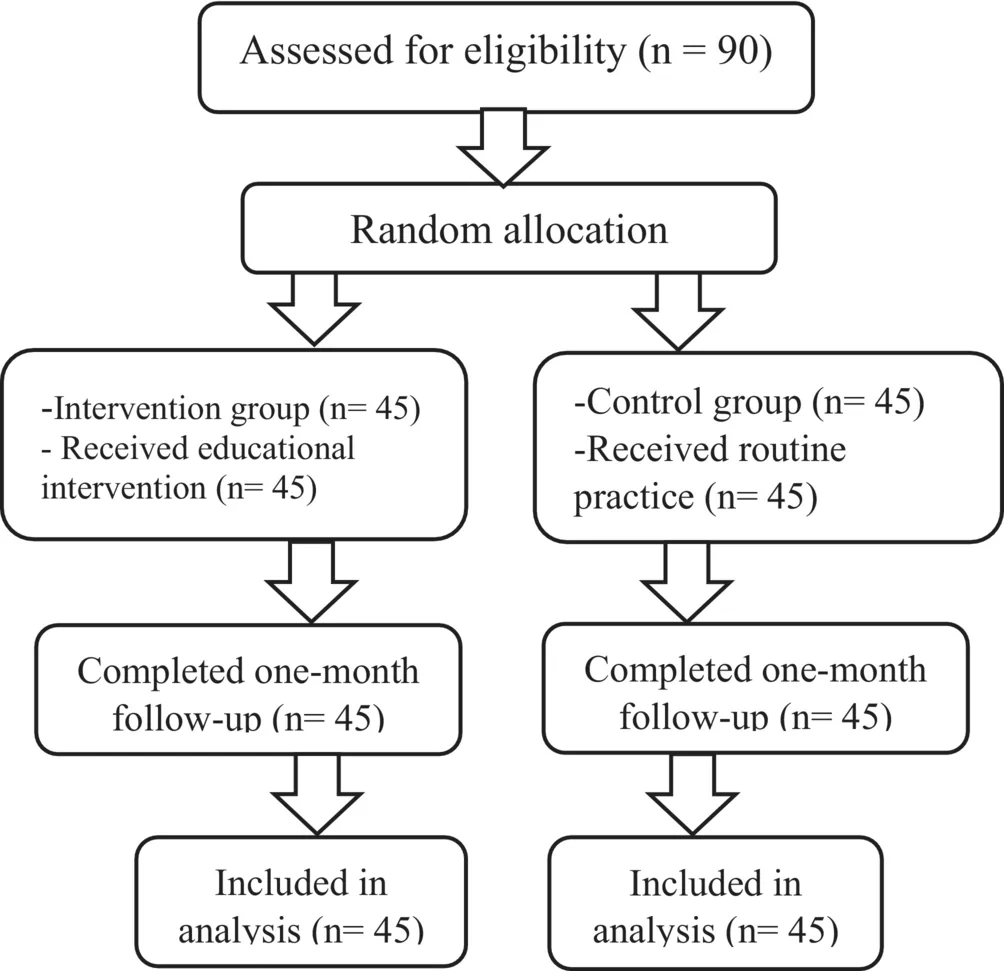
Flow of participants through the study.

Ninety eligible nurses were enrolled and randomly allocated to the intervention (*n* = 45) or control (*n* = 45) group. All participants completed the study, yielding a response rate of 100%, with no loss to follow‐up or missing outcome data. The flow of participants throughout the study is presented in Figure 1.

Baseline comparisons demonstrated no statistically significant differences between the two groups in demographic characteristics, including age, gender, marital status, educational level, employment status, occupational position, workplace ward, work shift, previous participation in aesthetic care workshops, previous participation in compassion competence workshops, information sources regarding aesthetic care, and years of work experience (all *p* > 0.05), indicating baseline comparability between groups.

The majority of participants in both groups were female, married, held a bachelor's degree, were officially employed, worked as clinical nurses, and performed rotational shifts. The mean age of participants was 39.64 ± 6.24 years in the intervention group and 38.53 ± 7.01 years in the control group. Mean work experience was 17.11 ± 6.61 years and 15.26 ± 5.86 years in the intervention and control groups, respectively (Table 2).

**TABLE 2:** Baseline demographic characteristics of participants in the intervention and control groups (*n* = 90).

Variable	Category	Intervention *n* (%)	Control *n* (%)	Test statistic	*p*
Gender	Male	5 (11.1)	11 (24.4)	*χ* ^2^ = 0.05	0.82
	Female	40 (88.9)	34 (75.6)		
Marital status	Single	3 (6.7)	7 (15.6)	*χ* ^2^ = 1.80	0.18
	Married	42 (93.3)	38 (84.4)		
Education	Bachelor's degree	41 (91.1)	39 (86.7)	*χ* ^2^ = 0.15	0.92
	Master's degree	4 (8.9)	6 (13.3)		
Employment status	Official	33 (73.3)	30 (66.7)	*χ* ^2^ = 3.24	0.35
	Contract	3 (6.7)	6 (13.3)		
	Company‐employed	7 (15.6)	4 (8.9)		
	Newly employed	2 (4.4)	5 (11.1)		
Position	Supervisor	2 (4.4)	0 (0.0)	*χ* ^2^ = 2.85	0.41
	Head nurse	14 (31.1)	11 (24.5)		
	Clinical nurse	29 (64.5)	34 (75.5)		
Ward	Management	2 (4.4)	0 (0.0)	*χ* ^2^ = 5.45	0.06
	Male ward	7 (15.6)	15 (33.3)		
	Female ward	36 (80.0)	30 (66.7)		
Working shift	Fixed	11 (24.4)	5 (11.1)	*χ* ^2^ = 2.73	0.09
	Rotational	34 (75.6)	40 (88.9)		
Previous aesthetic care workshop	Yes	3 (6.7)	4 (8.9)	*χ* ^2^ = 0.15	0.69
	No	42 (93.3)	41 (91.1)		
Previous compassion competence workshop	Yes	11 (24.4)	7 (15.6)	*χ* ^2^ = 1.11	0.29
	No	34 (75.6)	38 (84.4)		
Information source	During study	6 (13.3)	4 (8.9)	*χ* ^2^ = 7.64	0.10
	International conference	1 (2.2)	6 (13.3)		
	National conference	7 (15.6)	3 (6.7)		
	None	27 (60.0)	31 (68.9)		
Age (years)	Mean ± SD	39.64 ± 6.24	38.53 ± 7.01	*t* = −0.79	0.42
Work experience (years)	Mean ± SD	17.11 ± 6.61	15.26 ± 5.86	*t* = −1.39	0.16

*Note:* Data are presented as *n* (%) or mean ± SD. Chi‐square/Fisher's exact tests and independent *t*‐tests were used as appropriate.

### 3.2 Effect of the Intervention on Aesthetic Care

At baseline, there were no statistically significant differences between the intervention and control groups in the total aesthetic care score or any of its five dimensions (all *p* > 0.05). Following the intervention, nurses in the intervention group demonstrated significantly higher scores than those in the control group across all dimensions of aesthetic care, including compassionate commitment and competence, stress‐free care, humanistic attention to patients, patient satisfaction and comfort, and admirable commitment and competence (all *p* < 0.001). Likewise, the overall aesthetic care score increased markedly in the intervention group (81.02 ± 6.26) compared with the control group (46.24 ± 10.20) (*t* = −19.47, *p* < 0.001).

Within‐group analyses using paired *t*‐tests showed significant improvements from baseline to post‐intervention in all aesthetic care dimensions and in the total score among participants in the intervention group (all *p* < 0.001). In contrast, no statistically significant changes were observed in the control group (all *p* > 0.05). Effect sizes for post‐intervention between‐group comparisons were very large across all dimensions, indicating a substantial educational effect (Table 3).

**TABLE 3:** Comparison of aesthetic care scores between the intervention and control groups before and after the intervention.

Variable	Group	Pre‐Test Mean ± SD	Post‐Test Mean ± SD	Mean change	Cohen's *d*	Paired *t*	*p*
Compassionate commitment and competence	Intervention	8.46 ± 1.07	16.75 ± 2.05	8.28	5.06	−24.04	< 0.001
	Control	8.57 ± 2.17	9.13 ± 2.52	0.55	0.23	−1.13	0.26
Between‐group comparison		*t* = 0.30, *p* = 0.76	*t* = −15.68, *p* < 0.001	—	3.31	—	—
Stress‐free care	Intervention	8.84 ± 1.02	13.84 ± 2.16	5.00	2.97	−13.74	< 0.001
	Control	8.73 ± 1.35	9.31 ± 2.36	0.57	0.30	−1.52	0.13
Between‐group comparison		*t* = −0.43, *p* = 0.66	*t* = −9.43, *p* < 0.001	—	2.00	—	—
Humanistic attention to the patient	Intervention	8.57 ± 0.96	15.71 ± 2.24	7.13	4.14	−20.78	< 0.001
	Control	8.66 ± 1.33	8.95 ± 2.13	0.28	0.16	−0.75	0.45
Between‐group comparison		*t* = 0.36, *p* = 0.71	*t* = −14.64, *p* < 0.001	—	3.09	—	—
Patient satisfaction and comfort	Intervention	9.08 ± 1.12	18.73 ± 3.72	9.64	3.51	−17.37	< 0.001
	Control	9.20 ± 1.17	9.55 ± 2.38	0.35	0.18	−0.92	0.35
Between‐group comparison		*t* = 0.45, *p* = 0.64	*t* = −13.91, *p* < 0.001	—	2.93	—	—
Admirable commitment and competence	Intervention	8.28 ± 1.21	15.97 ± 2.02	7.68	4.61	−20.53	< 0.001
	Control	8.62 ± 1.00	9.28 ± 2.98	0.66	0.29	−1.45	0.15
Between‐group comparison		*t* = 1.41, *p* = 0.16	*t* = −12.44, *p* < 0.001	—	2.62	—	—
Overall aesthetic care	Intervention	43.26 ± 2.18	81.02 ± 6.26	37.75	8.05	−39.03	< 0.001
	Control	43.80 ± 3.48	46.24 ± 10.20	2.44	0.32	−1.59	0.11
Between‐group comparison		t = 0.87, *p* = 0.38	t = −19.47, *p* < 0.001	—	4.10	—	—

*Note:* Data are presented as mean ± SD. Within‐group comparisons were performed using paired *t*‐tests, between‐group comparisons using independent *t*‐tests, and effect sizes are reported as Cohen's d.

### 3.3 Effect of the Intervention on Compassion Competence

Baseline assessment revealed no statistically significant differences between the intervention and control groups in the overall compassion competence score or any of its subscales, including communication, sensitivity, and insight (all *p* > 0.05), indicating comparable levels of compassion competence before the intervention.

After completion of the intervention, the intervention group demonstrated significantly higher post‐test scores than the control group in all three compassion competence subscales. Specifically, communication increased to 25.91 ± 7.92 in the intervention group compared with 18.17 ± 3.47 in the control group (*t* = 5.99, *p* < 0.001). Likewise, sensitivity scores were significantly higher in the intervention group (19.46 ± 4.82) than in the control group (12.28 ± 2.38) (*t* = 7.71, *p* < 0.001). Similarly, insight scores increased significantly following the intervention (15.35 ± 4.57 vs. 10.24 ± 2.33; *t* = 6.67, *p* < 0.001).

The overall compassion competence score also increased significantly in the intervention group, reaching 60.73 ± 16.17 compared with 40.71 ± 4.54 in the control group (*t* = 7.99, *p* < 0.001).

Within‐group analyses demonstrated statistically significant improvements in communication, sensitivity, insight, and total compassion competence scores in the intervention group (all *p* < 0.001), whereas no significant changes were observed in the control group (all *p* > 0.05). The intervention produced statistically significant improvements in all compassion competence domains, with large effect sizes (Cohen's d = 1.26–1.68), indicating a substantial educational effect (Table 4). No adverse events related to the educational intervention were reported during the study period.

**TABLE 4:** Comparison of compassion competence scores between the intervention and control groups before and after the intervention.

Variable	Group	Pre‐Test Mean ± SD	Post‐Test Mean ± SD	Mean change	Cohen's *d*	Paired *t*	*p*
Communication	Intervention	17.93 ± 3.67	25.91 ± 7.92	7.97	1.29	−6.12	< 0.001
	Control	17.08 ± 2.87	18.17 ± 3.47	1.08	0.34	−1.68	0.09
Between‐group comparison		*t* = 1.21, *p* = 0.22	*t* = 5.99, *p* < 0.001	—	1.26	—	—
Sensitivity	Intervention	12.68 ± 2.53	19.46 ± 4.82	6.77	1.76	−7.60	< 0.001
	Control	12.40 ± 2.22	12.28 ± 2.38	−0.11	0.05	0.24	0.80
Between‐group comparison		*t* = −0.03, *p* = 0.97	*t* = 7.71, *p* < 0.001	—	1.62	—	—
Insight	Intervention	10.17 ± 2.38	15.35 ± 4.57	5.18	1.42	−6.17	< 0.001
	Control	9.86 ± 2.38	10.24 ± 2.33	0.38	0.16	−0.80	0.42
Between‐group comparison		*t* = 0.61, *p* = 0.53	*t* = 6.67, *p* < 0.001	—	1.40	—	—
Total compassion competence	Intervention	40.80 ± 5.15	60.73 ± 16.17	19.93	1.66	−7.47	< 0.001
	Control	39.35 ± 4.28	40.71 ± 4.54	1.35	0.30	−1.54	0.12
Between‐group comparison		*t* = 1.44, *p* = 0.15	*t* = 7.99, *p* < 0.001	—	1.68	—	—

*Note:* Data are presented as mean ± SD. Within‐group comparisons were performed using paired *t*‐tests, between‐group comparisons using independent *t*‐tests, and effect sizes are reported as Cohen's d.

## 4 Discussion

This study investigated the effect of an aesthetics‐based nursing care educational program on aesthetic care and compassion competence among psychiatric nurses. The findings demonstrated that the intervention significantly improved both outcomes compared with the control group. These findings suggest that educational strategies emphasizing reflective learning, lived experiences, and active participation may facilitate the internalization of humanistic care concepts, enhance sensory awareness, and strengthen nurses' appreciation of the artistic and ethical dimensions of nursing practice (Lv et al. 2025; Yang et al. 2022).

The observed improvements in aesthetic care and compassion competence may be related to the interactive and reflective nature of the educational intervention. The use of group discussions and scenario‐based learning may have encouraged nurses to reflect on their clinical experiences and consider the emotional and interpersonal aspects of patient care. Furthermore, sharing lived experiences and participating in reflective activities may have enhanced professional self‐awareness and sensitivity toward patients' needs. These educational strategies are consistent with the principles of aesthetics‐based nursing care, which emphasize conscious presence, empathy, creativity, and attention to patients' subjective experiences (Betriana et al. 2022; Hua et al. 2025). Therefore, the combination of reflective learning and experiential activities may have contributed to the development of more compassionate and aesthetically oriented nursing practices.

Khooshab et al. (2024) identified aesthetic experience of care as a means to foster a more humane and empathetic connection with patients Bachtiar et al. (2023) demonstrated that programs targeting caring behaviours significantly improve nurses' respect, attentiveness, and ability to provide comfort and reassurance, thereby improving patient satisfaction (Bachtiar et al. 2023). Hua et al. (2025) noted that aesthetic‐based care, as a manifestation of the art of nursing, transcends technical skills and highlights the importance of conscious presence, creativity, situational sensitivity, and the nurse's perceptual capacities (Hua et al. 2025). Li et al. (2025) demonstrated that aesthetics facilitates the conversion of intuitive cognition into artistic behaviour, allowing nurses to better meet genuine patient needs (Li et al. 2025). Together, these findings support the present results and suggest that aesthetics‐based education enhances both the artistic and relational dimensions of nursing care.

The study found that the aesthetics‐based educational intervention significantly enhanced the CC of nurses in the intervention group. This efficacy underscores the role of structured training in refining the humane and emotional competencies essential for empathetic and compassionate care. The results align with previous studies indicating that aesthetics‐based nursing education correlates with the compassionate competence characteristics of nurses (Fesharaki and Radmehr 2020; Shahmohammadi et al. 2024). For instance, Lee and Seo (2022) noted that nurses with positive caring behaviours not only provide higher quality nursing services but also display superior CC (Lee and Seo 2022). Additionally, Hong and Han (2021) identified communication skills development as a practical approach to augmenting nurses' caring behaviours (Hong and Han 2021). The observed improvement may be explained by the use of authentic and simulated learning contexts that encouraged nurses to reflect on their clinical experiences. According to Salehi et al.'s (2025) study, a lack of organizational support and adequate training can result in burnout and emotional exhaustion despite a nurse's desire for emotional connection (Salehi et al. 2025). Conversely, a compassion deficit may hinder a nurse's ability to deliver the true essence of care (Li et al. 2025). Aesthetic‐based care education establishes a foundation for improved therapeutic communication and patient satisfaction, while simultaneously reinforcing the nurse's emotional resilience against burnout through the cultivation of compassion. These findings suggest that compassion competence can be strengthened through structured educational interventions rather than being regarded solely as an innate personal characteristic.

The findings also have important implications for nursing education and clinical practice. Incorporating aesthetics‐based educational strategies into undergraduate nursing curricula, orientation programs, and continuing professional development initiatives may strengthen nurses' ability to establish therapeutic relationships and deliver compassionate care. In psychiatric settings, where trust, communication, and emotional support are essential components of effective nursing practice, such educational approaches may contribute to improved patient experiences, enhanced professional satisfaction among nurses, and a more humanistic care culture (Hong and Han 2021; Lee and Seo 2022). Healthcare managers and nursing educators may therefore consider integrating aesthetics‐based education into routine staff development programs, particularly in psychiatric settings.

## 5 Conclusion

The study's findings revealed that aesthetic‐based care training, as a new educational paradigm, has the potential to improve psychiatric nurses' understanding of CC and aesthetic care. The findings underscore that enhancements in care quality are not exclusively reliant on technological or technical interventions; rather, they are fundamentally grounded in humane communication, compassion, and aesthetic disposition. The current study offers empirical evidence bridging the gap between aesthetic care theories and clinical practice. By integrating theoretical knowledge with practical experience—utilizing case studies and reflective group discussions—this educational program enhances nurses' competence in both compassion and aesthetics. As a result, nurses are better equipped to deliver quality care and make informed decisions. The results of this study serve as a foundation for developing educational interventions for nursing students and nurses within a comprehensive mental health care system.

## 6 Limitations

This study has several limitations that should be considered when interpreting the findings. First, the study was conducted in a single psychiatric hospital, which may limit the generalizability of the findings to other healthcare settings and populations. Second, outcomes were assessed only 1 month after completion of the intervention. Although this follow‐up period allowed evaluation of the short‐term retention of the educational effects, it does not provide evidence regarding their long‐term sustainability. Future studies with longer follow‐up periods are therefore warranted.

Third, all outcome measures were based on self‐reported questionnaires, making the findings susceptible to social desirability bias, recall bias, and response expectancy. Participants' awareness of the educational intervention may also have influenced their responses, potentially contributing to the large observed effect sizes. Fourth, neither participants nor researchers were blinded to group allocation, which increases the risk of performance and assessment bias. In addition, the Hawthorne effect cannot be excluded, as participants knew they were taking part in an educational intervention study.

Although participants were randomly allocated to study groups, several baseline demographic variables differed between groups. However, no statistically significant differences were observed in the baseline scores of the primary outcome measures (aesthetic nursing care and compassion competence), suggesting that the post‐intervention improvements were unlikely to be explained by initial differences in the study outcomes. Nevertheless, residual confounding cannot be completely ruled out.

Furthermore, intervention fidelity and participants' adherence to the educational program were not formally evaluated. Finally, this study focused exclusively on nurse‐reported outcomes and did not assess patient‐centered outcomes such as patient satisfaction, therapeutic alliance, quality‐of‐care indicators, or clinical recovery. Future multicenter studies with larger samples, longer follow‐up periods, objective outcome measures, assessment of intervention fidelity, adjustment for potential confounding variables, and inclusion of patient‐related outcomes are recommended to strengthen the evidence.

## Author Contributions

**Donya Balideh:** data curation, writing – original draft, investigation, resources, writing – review and editing. **Jamileh Farokhzadian:** conceptualization, supervision, writing – original draft, writing – review and editing. **Sakineh Miri:** writing – review and editing, writing – original draft, conceptualization. **Sedigheh Khodabandeh Shahraki:** conceptualization, supervision, writing – review and editing, writing – original draft, investigation, project administration. **Mohadeseh Motamed‐Jahromi:** formal analysis, writing – review and editing, writing – original draft, methodology, resources.

## Funding

The authors have nothing to report.

## Ethics Statement

The Ethics Committee of Kerman University of Medical Sciences approved the study (IR.KMU.REC.1403.313) (Reg. No. 403000441). At the request of the ethics committee, the study was conducted in accordance with the Declaration of Helsinki and the Committee on Publication Ethics (COPE). Each participant signed an informed consent during the research process and was informed of the study objectives, the method of cooperation, the method of data collection and recording, the role of the researcher and the participants, and their voluntary participation and privacy.

## Conflicts of Interest

The authors declare no conflicts of interest.

## Supporting information


**File S1:** TREND checklist completed for the reporting of the present nonrandomized study.

## Data Availability

The data are available upon request to the corresponding author after signing appropriate documents in line with ethical application and the decision of the Ethics Committee.
